# Facial Thread Lifting Complications

**DOI:** 10.1111/jocd.16745

**Published:** 2025-01-06

**Authors:** Kyu‐Ho Yi, Soo Yeon Park

**Affiliations:** ^1^ Division in Anatomy and Developmental Biology, Department of Oral Biology, Human Identification Research Institute, BK21 FOUR Project Yonsei University College of Dentistry Seoul Korea; ^2^ Maylin Clinic (Apgujeong) Seoul Korea; ^3^ Made‐Young Plastic Surgery Clinic Seoul Korea

**Keywords:** dimpling, facial anatomy, facial palsy, facial retaining ligament, hematoma, thread lifting

## Abstract

**Background:**

Thread lifting is a minimally invasive technique for addressing facial aging and skin laxity. Despite its popularity, it carries risks of complications ranging from minor bruising to severe structural injuries. Comprehensive understanding of these complications is vital for optimizing outcomes.

**Aims:**

To analyze the complications associated with facial thread lifting, provide insights into their underlying mechanisms, and propose effective management strategies to minimize risks.

**Patients/Methods:**

A comprehensive review of complications from thread lifting was conducted, focusing on patient outcomes and procedural challenges. Data from clinical studies, case reports, and expert recommendations were analyzed to identify common and severe complications. Advanced techniques, such as ultrasound‐guided procedures, were evaluated for their efficacy in preventing complications.

**Results:**

Common complications included bruising, pain, swelling, bleeding, and dimpling, often arising from improper technique or anatomical factors. Severe issues such as infection, granuloma, thread migration, nerve damage, and parotid gland injuries were less frequent but posed significant challenges. Management strategies included massage, saline or filler injections, botulinum toxin, and surgical interventions for persistent or severe cases. Ultrasound‐guided techniques demonstrated efficacy in reducing risks, particularly for glandular and vascular injuries.

**Conclusions:**

While thread lifting offers a promising alternative to surgical facelifts, its success depends on precise technique, anatomical knowledge, and effective complication management. Preventive measures, including preoperative planning, proper thread placement, and patient‐specific customization, are crucial. Future advancements in imaging technologies and thread materials will likely enhance safety and efficacy, ensuring better patient satisfaction and outcomes.

## 1 Introduction

As individuals enter their middle and later years, the signs of aging become increasingly evident, with deeper wrinkles and more noticeable sagging skin. This growing visibility of aging features drives a significant demand for facelift surgeries. These procedures are designed to tighten and remove excess skin, effectively addressing the heightened laxity of both the skin and underlying tissues [1].

Thread lifting procedures often result in immediate adverse events such as bruising and swelling, which fortunately usually do not lead to long‐term complications. These adverse events can be minimized by using a gentle, deliberate approach during the procedure, preferably employing cannulas instead of needles. The current focus is on thoroughly examining cases where adverse events significantly impact aesthetic outcomes or may lead to lasting complications, rather than those that are easily manageable [2, 3].

Thread lifting procedures may result in various short‐term complications, including bruising, pain, swelling, bleeding, and hematoma formation. Other issues can include dimpling, skin irregularities, and alterations in facial contour [4]. Additionally, neurosensory effects such as tension, numbness, and itching may occur. More severe complications encompass infection, inflammation, abscess formation, thread extrusion, subcutaneous induration, granuloma formation, and potential impacts on adjacent structures, including facial nerves, the parotid gland, and its duct [5].

Especially pertinent are the common complications associated with thread lifting, such as dimpling, protrusion, skin penetration from skin tagging, parotitis, and nerve damage, many of which stem from anatomical factors. A notable example is the frequent appearance of depressions or dents in the skin, resembling dimples, particularly prominent in the cheek area following cog thread lifting procedures. These dimples can appear immediately after the procedure or gradually over several days to weeks. While some cases may resolve on their own, patients often experience discomfort, prompting them to seek medical care [6, 7]. Accurate placement of threads within the subcutaneous tissue layer is essential to minimize the risk of dimpling, with slight hypercorrection recommended during the procedure, especially in older patients, where minor contour irregularities are more likely to occur.

Physicians, particularly those encountering this complication for the first time, may feel uneasy. However, it's important to understand that dimples can be effectively corrected through manual molding with the fingers. The probability of dimple formation and the suitability of post‐procedural treatment methods can vary depending on the type of thread used and its insertion site. This highlights the importance of pre‐treatment discussions with patients about these potential outcomes and their management. Such proactive communication helps build a strong and positive patient‐provider relationship, ensuring informed decision‐making and comprehensive care [8].

This article aims to provide a comprehensive analysis of these complications, offering insights into their management and highlighting best practices to optimize patient outcomes and safety.

## 2 Dimples

**FIGURE 1:**
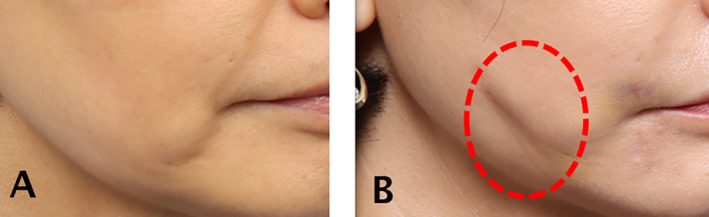
Placing threads too near the skin can lead to dimples; therefore, it is crucial to insert them at the proper depth. Additionally, the tension applied when trimming the threads affects dimple formation. Illustrated before the procedure (A) and after the procedure (B).

The frequency of complications following thread lifting procedures varies slightly across different journals. Generally, dimple formation is considered the most common complication that requires correction. To understand the process behind dimple formation after thread lifting, it is crucial to recognize the variation in the density of fibrotic tissues within the fat layer. The area where the cogs securely anchor post‐thread insertion is characterized by a specific distribution of fibrotic tissues within the fat tissues, ensuring that the cog remains in place after lifting and yields a favorable outcome. However, an excessive density of fibrotic tissues within the fat layer can hinder the passage of a cannula or needle. Additionally, when cogs anchor firmly in unintended positions, dimples can easily form. Using barbed threads for lifting can cause slight dimpling if the barbs catch near the skin. Thicker threads or products with cones have a higher likelihood of causing dimples. Threads placed too close to the skin during the procedure can also result in dimples, so it is crucial to insert threads at an appropriate depth (Figure 1). The amount of tension applied to the threads when cutting them also affects dimple formation [9].

Slight dimples that appear immediately after the procedure often become more natural as swelling subsides. Mild dimples can sometimes be alleviated with massage. Since initial tumescent effects or swelling might obscure dimples, it is important for patients to return for a follow‐up visit within 1 or 2 days after the procedure to check for any complications and address symptoms that may arise as swelling decreases. If the barbs remain engaged with the tissues for too long, fibrosis between the threads and tissues can lead to persistent dimples. If massage or molding does not resolve a skin dimple, injecting saline into the sunken area followed by massage or molding can help release the barbs from the skin or soft tissue more easily. Alternatively, injecting a soft filler to smooth the surface can enhance the skin's appearance [10].

**FIGURE 2:**
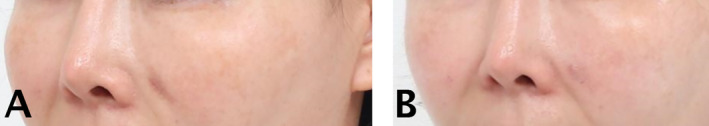
Placing volume threads too shallowly can cause them to grip the dermis, making them palpable. In such cases, the threads may become scar tissue and persist. Shown before the procedure (A) and after thread removal (B).

Volume threads placed too shallowly can also cause the threads to grip the dermis, making their presence palpable. In such cases, the threads may become scar tissue and persist (Figure 2). Immediate removal is an option, or if the patient prefers not to have the threads removed, subcision followed by the injection of free hyaluronic acid (HA) to catalyze the degradation of the threads can be attempted. Follow‐up and secondary treatment include an immediate postoperative check and a delayed check between 2 weeks and 1 month after the procedure [11, 12].

## 3 Bulging (Contour Irregularity)

After inserting bidirectional threads, excessive pulling can cause bulging in the middle area, disrupting the facial contour. This is particularly concerning in the Asian population, where pronounced cheekbones are often avoided, as bidirectional thread lifting may accentuate the cheekbones. Therefore, it is essential to carefully choose the direction of bidirectional threads or consider using multidirectional or fixation threads to distribute the lifting effect more naturally.

**FIGURE 3:**
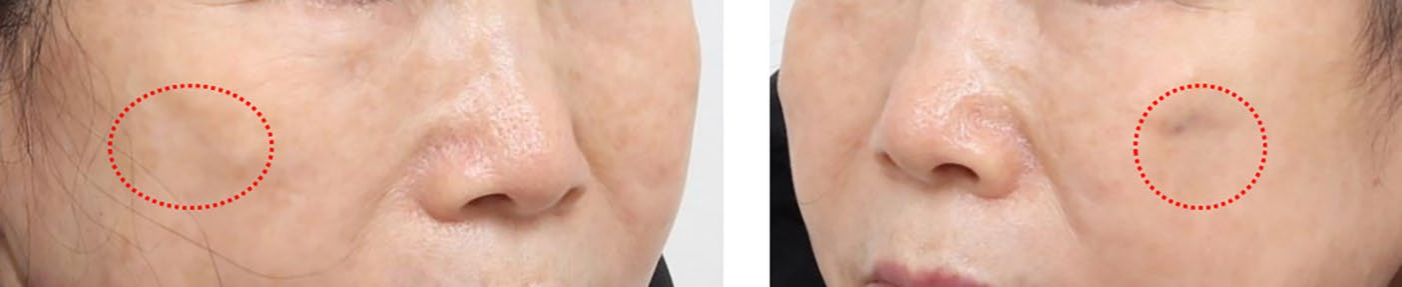
Contour irregularities can occur, and while massage can provide some improvement, persistent issues may necessitate thread cutting or removal.

When long threads are used and fixed at the temple, contour irregularities may occur. While some improvement can be achieved through massage, similar to treating dimples, persistent issues may require thread cutting or removal (Figure 3).

**FIGURE 4:**
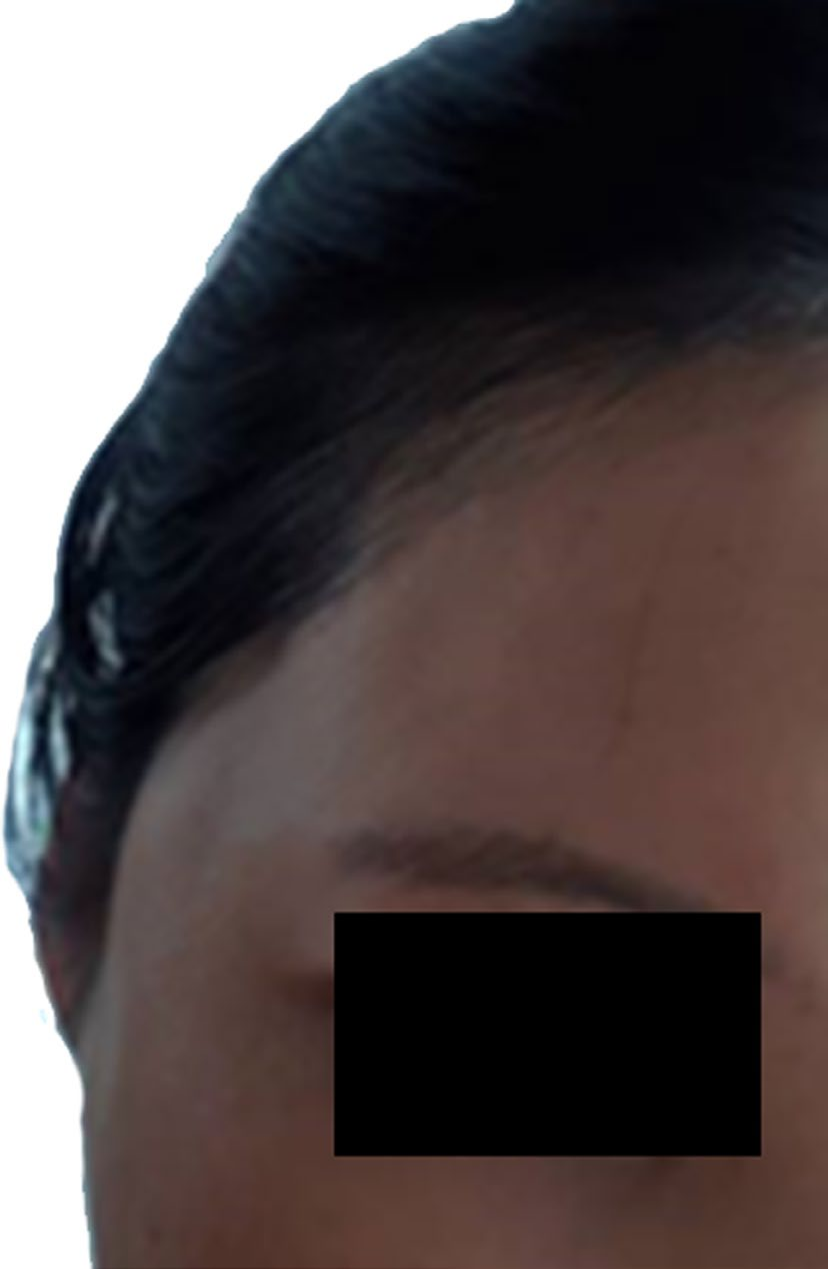
There has been a recent increase in inserting threads in the reverse direction (reverse vector) towards the temple. If the thread is not placed between the superficial and deep temporal fascia and is inserted too superficially, the tissue may protrude during chewing movements. This condition may improve over time, but severe cases might benefit from botulinum toxin injections into the temporalis muscle.

Recently, there has been an increase in inserting threads in the reverse direction (reverse vector) towards the temple. If the thread is not inserted between the superficial temporal fascia and deep temporal fascia and is placed too superficially, the tissue may protrude during chewing movements. Although this condition may improve over time, severe cases may benefit from botulinum toxin injections into the temporalis muscle (Figure 4).

## 4 Migration

**FIGURE 5:**
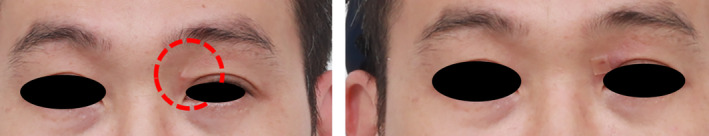
In one case of eyebrow lifting, the thread did not anchor well at the fixation point, causing it to migrate down to the eyelid. Shown before the procedure (A) and after thread removal (B).

**FIGURE 6:**
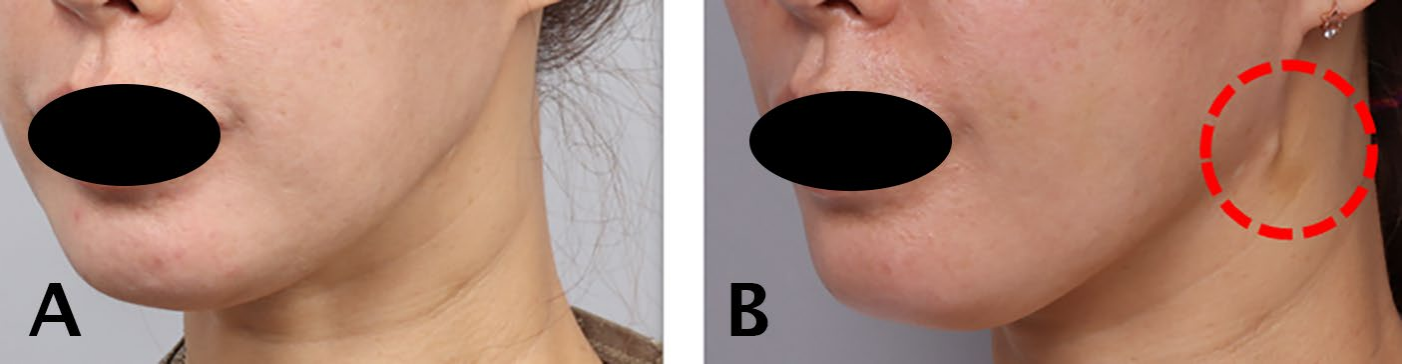
Another case involved using a double‐arm needle thread for a hammock technique to address a double chin. If the thread fails to secure properly to the dense part of the cervical fascia, thread migration can occur, as seen in this patient.

Thread migration is a common occurrence after thread lifting procedures and can be attributed to several potential causes, including the use of unstable threads that may break or fail to secure properly at fixation points. For example, in one patient who underwent eyebrow lifting, the thread did not anchor well at the fixation point, causing it to migrate down to the eyelid (Figure 5). This could result from using unstable threads or improper anchoring. Another case involved using a double‐arm needle thread to perform a hammock technique for a double chin. If the thread fails to secure properly to the dense part of the cervical fascia, as in this patient's case, thread migration can occur (Figure 6). Ensuring secure fixation at critical anatomical landmarks is essential to prevent thread migration, particularly in high‐movement areas such as the mouth and forehead, where botulinum toxin can be applied to reduce muscle activity and further stabilize thread positioning.

## 5 Pre‐Protrusion and Protrusion

**FIGURE 7:**
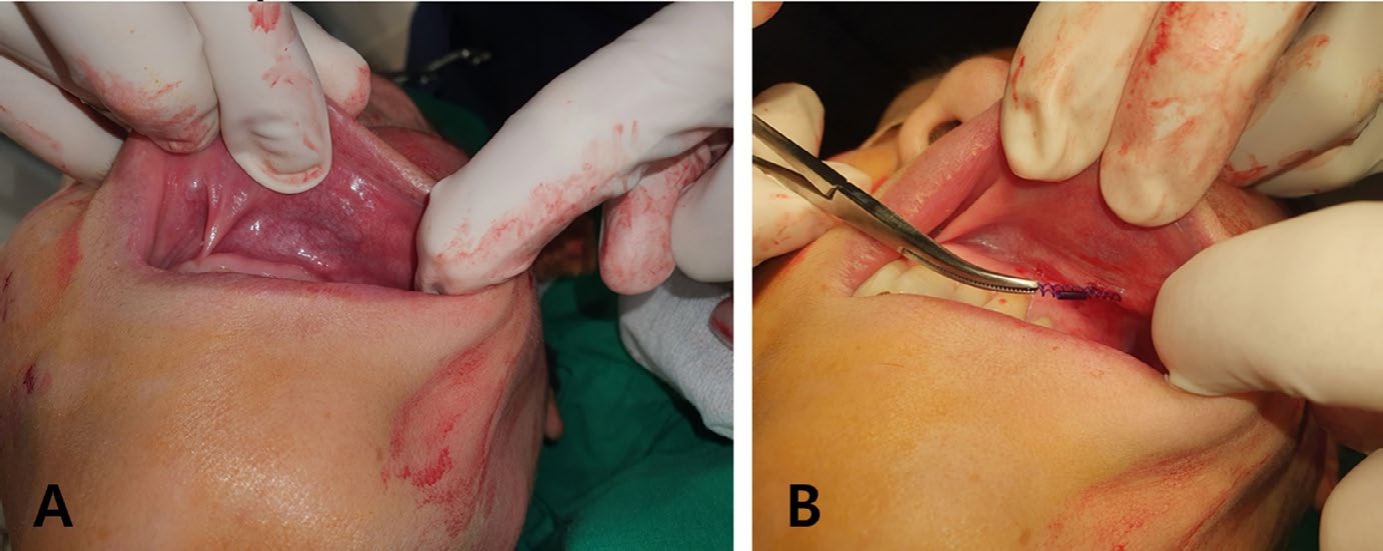
If a thread is on the verge of protruding (pre‐protrusion), create a small hole with a needle and carefully extract the thread. Shown before the correction (A) and during thread removal (B).

**FIGURE 8:**
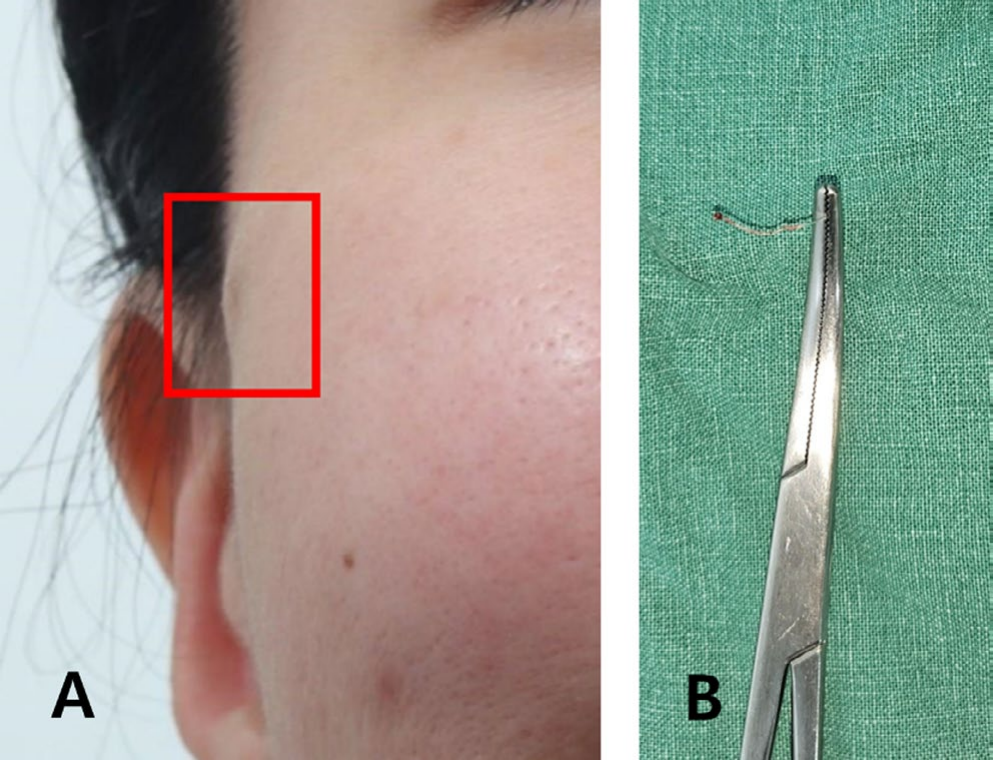
When the thread only protrudes during specific facial expressions, have the patient make that expression (A), then locate and remove the thread end using fine Castroviejo forceps (B).

**FIGURE 9:**
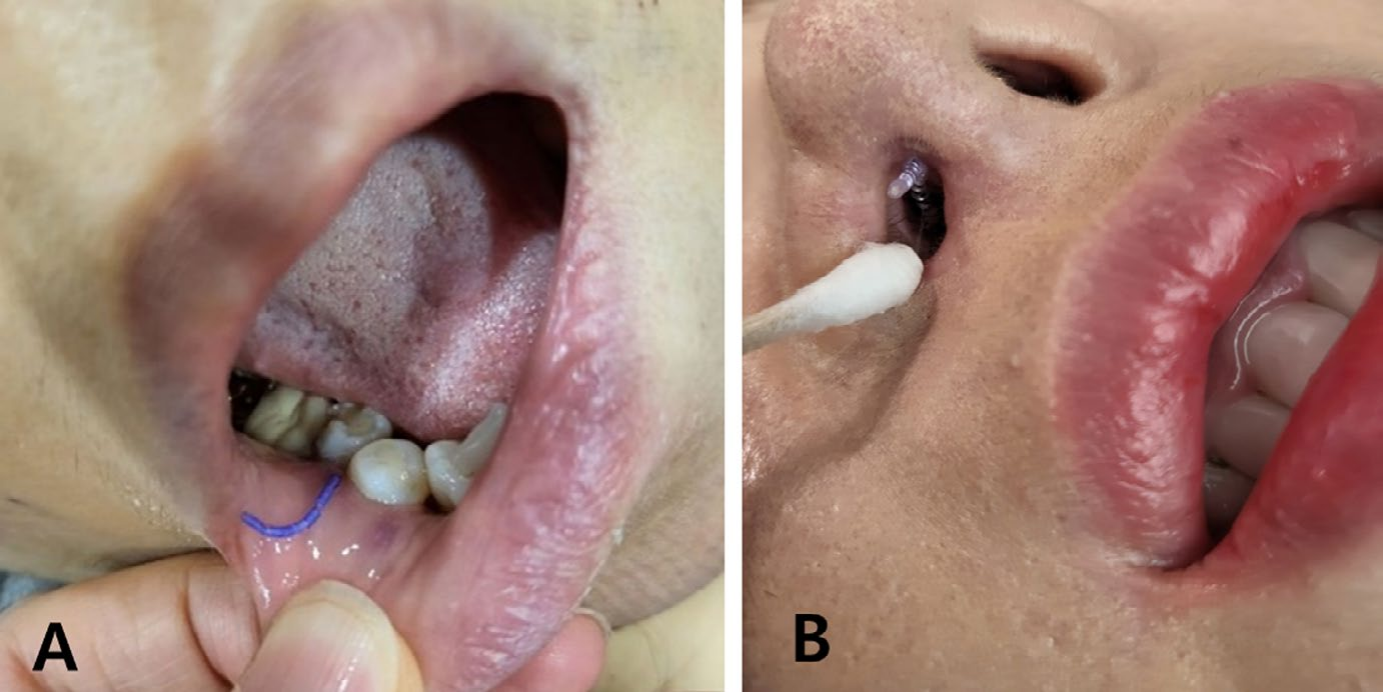
Threads may protrude from the skin or mucosa due to factors such as body positioning or continuous movement, resulting in extrusion. Shown in (A) and (B).

If a thread is on the verge of protruding (pre‐protrusion), make a small hole with a needle and carefully extract the thread (Figure 7). When the thread only protrudes during specific facial expressions, have the patient make that expression, then locate and remove the thread end using fine Castroviejo forceps (Figure 8). Additionally, threads may protrude from the skin or mucosa due to factors such as body positioning or continuous movement, resulting in extrusion (Figure 9). Thread migration towards the skin's surface increases the likelihood of extrusion, as displaced threads are more prone to protruding, making secure placement and stabilization critical in preventing this complication.

## 6 Thread Palpation

**FIGURE 10:**
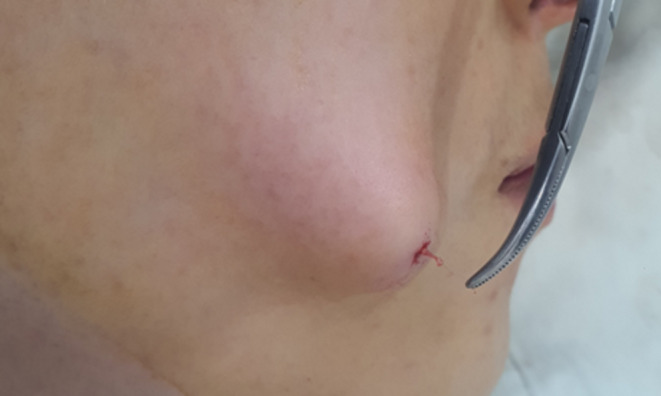
When the end of a thread can be felt on the surface, known as thread palpation, attempting to grasp the thread from a point beyond the distal end with forceps can cause it to slip deeper. Instead, make an incision slightly proximal to the thread's end. For very thin threads, such as those around 3‐0 in size, pulling directly may cause them to break. Instead, use fine Castroviejo forceps to gently find the middle section of the thread and carefully pull it proximally to remove as much of the thread as possible.

When the end of a thread can be felt on the surface, this is known as thread palpation. If you try to grasp the thread with forceps from a point beyond the distal end, the thread tends to slip deeper. Therefore, make an incision slightly proximal to the very end of the thread. For very thin threads, such as those around 3‐0 in size, pulling them directly might cause them to break. Instead, use fine Castroviejo forceps to gently find the middle section of the thread, then carefully pull it like a tug‐of‐war to move proximally and remove as much of the thread as possible (Figure 10).

## 7 Granuloma

**FIGURE 11:**
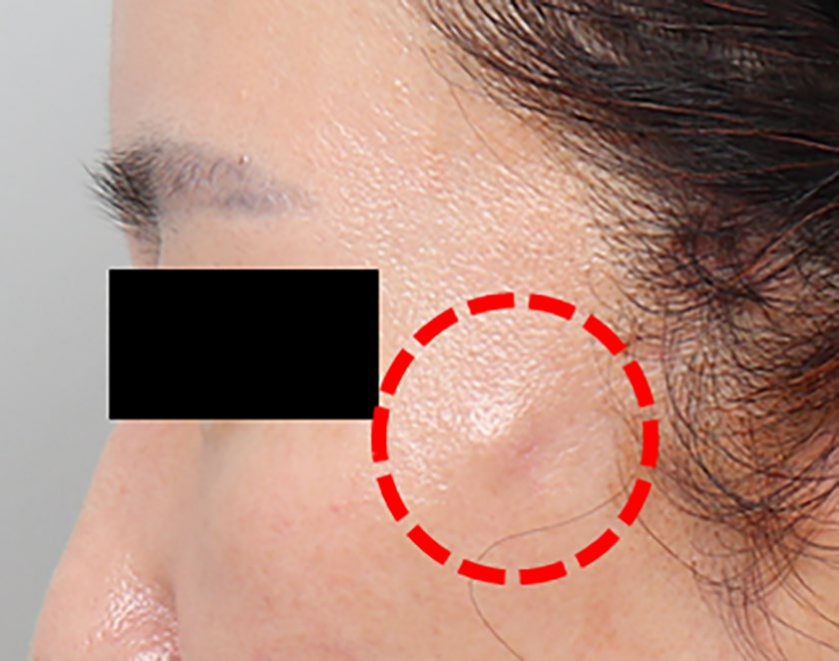
If the thread is inserted too superficially, it can continuously irritate the dermis or catch fibers like gauze lint on its barbs.

**FIGURE 12:**
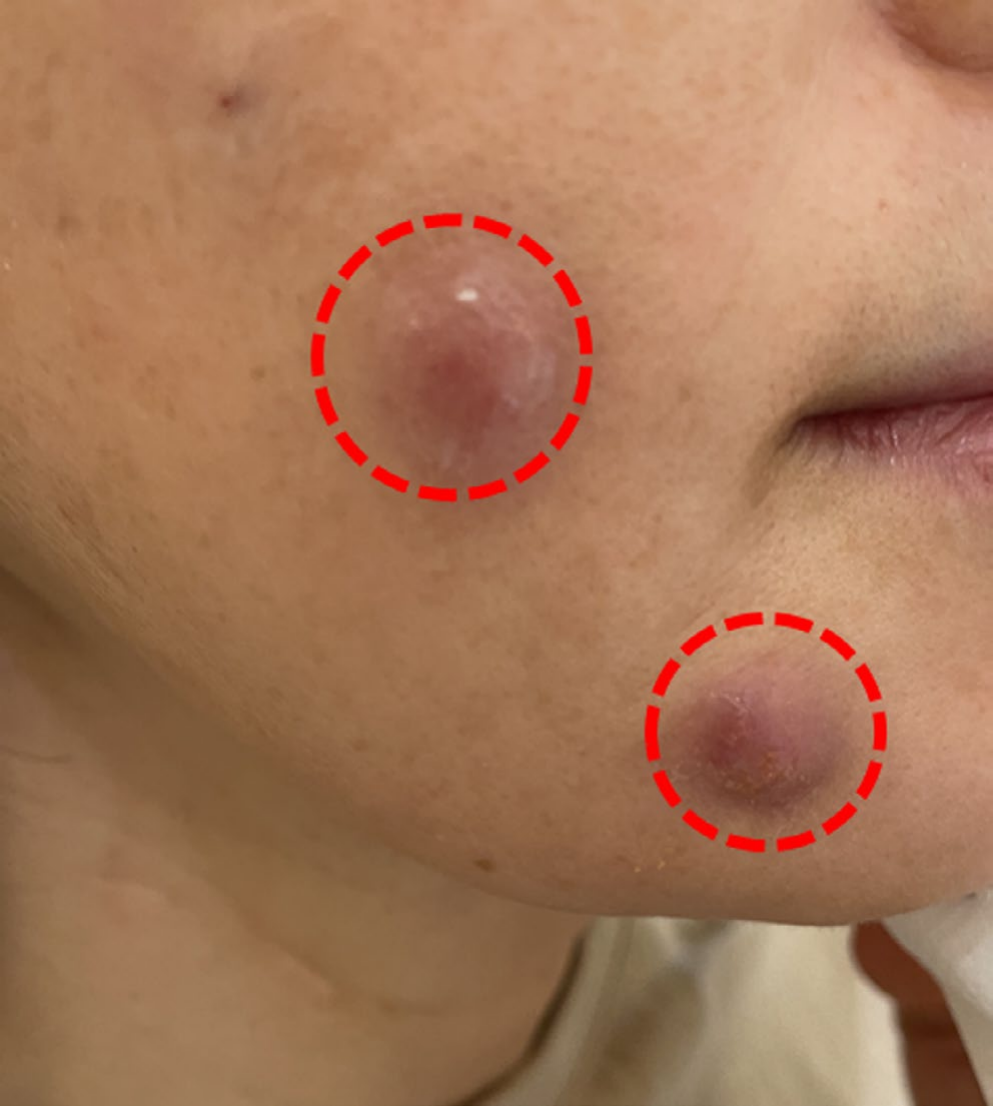
Improper skin sterilization can lead to an inflammatory reaction, potentially causing granuloma formation. These granulomas can present cosmetic issues and pose a risk of secondary infection from skin‐resident bacteria.

If the thread is inserted too superficially, it can continuously irritate the dermis or catch fibers like gauze lint on its barbs (Figure 11) [13, 14, 15, 16]. Additionally, if the skin is not properly sterilized, an inflammatory reaction may occur, potentially leading to granuloma formation. These granulomas can pose not only cosmetic issues but also a risk of secondary infection from skin‐resident bacteria (Figure 12). In such cases, the primary treatment involves reducing the inflammation and subsequently removing the thread. If the patient does not wish to remove the thread but the tissue reaction persists, localized injections of steroids or 5‐FU can help mitigate the inflammatory response. Granuloma formation is commonly linked to superficial thread placement, as threads positioned too close to the dermis can cause localized trauma, triggering an inflammatory or granulomatous response.

## 8 Hyperpigmentation

Hyperpigmentation can occur at the insertion points of lifting threads. In this patient's case, pigmentation developed in front of the sideburns and on the cheeks. Although it tends to fade over time, using treatments like picolaser can accelerate the process.

## 9 Residual Cannula

**FIGURE 13:**
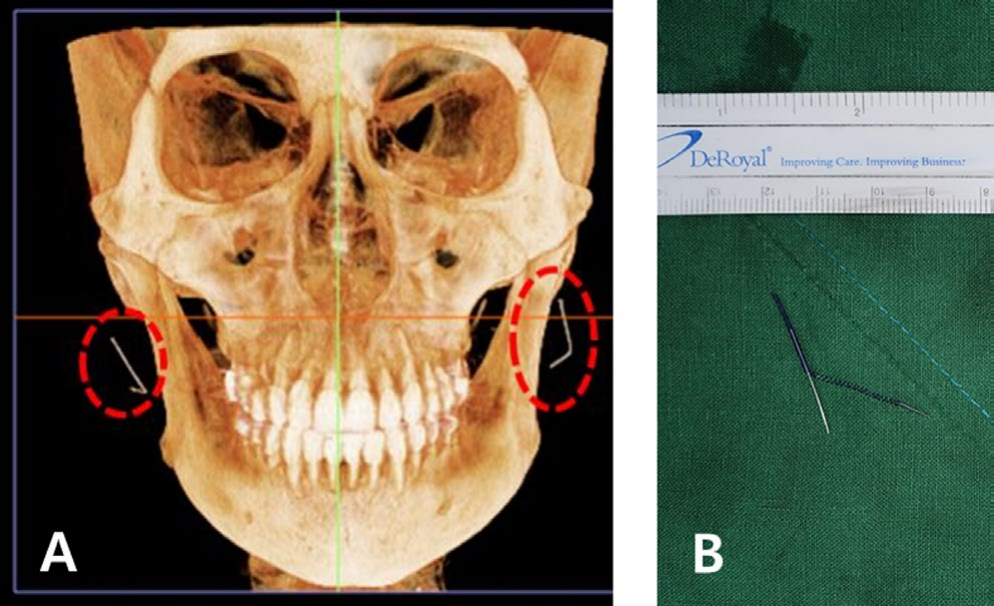
When using cannula‐integrated threads, there is a possibility that the cannula and hub (handle part) can separate, leaving the cannula embedded inside. Shown in a computed tomography image (A) and during cannula removal (B).

When using cannula‐integrated threads, there is a potential risk of the cannula detaching from the hub and becoming embedded in the tissue, as shown in Figure 13. This issue is particularly prevalent with aluminum cannulas, which may break off and remain lodged with the thread. For instance, in the case of a Jamber 27G volumizing thread, the removal of a broken cannula often requires a surgical incision.

**FIGURE 14:**
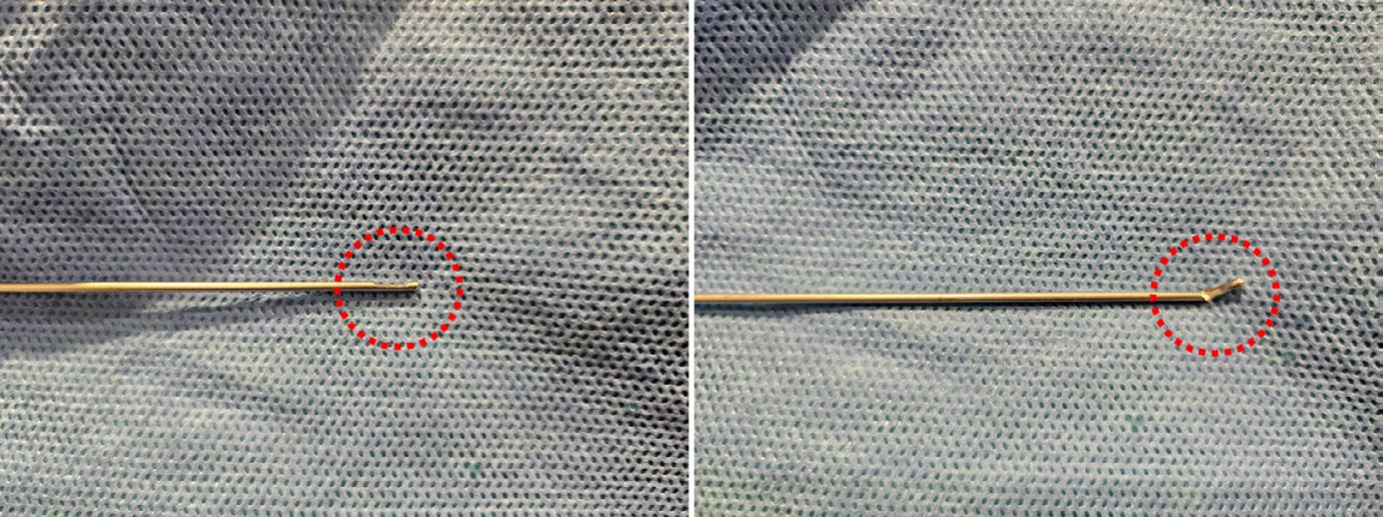
Inserting the cannula into particularly dense or fibrous tissue can apply excessive force, causing the tip to crumple or deform. Upon withdrawal, the deformed tip may break off, leaving part of the cannula embedded in the tissue.

In procedures involving integrated cannula and PDO cog lifting threads, the cannula tip is designed with a slight opening where the thread is exposed. While this design facilitates insertion, it also presents a risk due to the inherent weaknesses in the aluminum material, particularly at the tip (Figure 14). There are two primary scenarios where the cannula tip might break during the procedure:

1. Manufacturing Defects: If the cannula is defective, the tip area may be weakened, leading to bending or breakage during use. In such cases, the issue should be reported to the manufacturer for corrective action, as it constitutes a product defect rather than a procedural complication.
2. Excessive Force in Dense Tissue: When the cannula is inserted into particularly dense or fibrous tissue, excessive force can cause the tip to crumple or deform. Upon withdrawal, the deformed tip may break off, leaving part of the cannula embedded in the tissue. This scenario typically reflects operator error, where improper technique or excessive force is applied, rather than a product defect.

These scenarios underscore the importance of inspecting the cannula for any potential defects before use and employing a cautious technique when encountering resistance during insertion. Proper handling and awareness of these risks can significantly reduce the likelihood of cannula breakage, ensuring both the safety and effectiveness of the procedure.

## 10 Infection

Furthermore, it is crucial to consider the risk of infection when discussing complications related to thread‐lifting procedures. Signs of inflammation, such as redness, warmth, and swelling along the path of the inserted threads, can indicate an infection in the skin. To reduce these risks, it is advised to administer prophylactic antibiotics for about 3–5 days following the procedure. Additionally, thorough disinfection with betadine before the procedure is essential. Although many practitioners likely follow these protocols, it is important to emphasize their significance. To minimize infection risks, strict adherence to aseptic techniques during the procedure is essential, along with comprehensive patient education on post‐procedural care, including avoiding face washing or pet interaction within the first 48 h to prevent contamination at puncture sites.

## 11 Nerve Damage

The facial nerve's temporal, zygomatic, buccal, and marginal mandibular branches emerge from the parotid gland and run anteriorly beneath the superficial muscular aponeurotic system (SMAS). These branches of the facial nerve are positioned deep laterally but become more superficial as they progress medially, with smaller branches innervating the facial muscles. Among these, the temporal branch exits the parotid gland by penetrating the parotidomasseteric fascia and travels upward across the zygomatic arch, covered by the innominate fascia. It penetrates the innominate fascia approximately 1.5–3 cm above the zygomatic arch and runs just beneath the superficial temporal fascia. The temporal branch follows Pitanguy's line, which extends from a point 0.5 cm below the tragus to a point 1.5 cm above the lateral eyebrow. It crosses the middle third of the zygomatic arch, located 1.8 cm from the posterior end and 2 cm from the anterior end of the arch. The temporal branch is more vulnerable to damage near the parotid gland, where the nerve trunk is thicker and less protected, making it more susceptible to injury than the smaller distal branches.

The greater auricular nerve (GAN) is a significant sensory nerve responsible for providing sensation to areas such as the parotid gland, skin over the mastoid process, and the external ear. The GAN originates from the cervical plexus, primarily from the C2 and C3 nerve roots, and courses vertically across the sternocleidomastoid (SCM) muscle, approximately 6.5 cm below the lower edge of the external auditory meatus. The GAN runs superficially in the neck, positioned posterior to the platysma muscle, which renders it particularly vulnerable to injury during procedures like thread lifting, especially due to its lack of protective coverings.

Nerve damage to the GAN during thread lifting can occur due to improper technique, such as using sharp needles or applying excessive tension during thread placement. The GAN is particularly susceptible to injury because it traverses the sub‐platysmal fat layer, a region frequently targeted in thread lifting procedures aimed at enhancing neck and facial contours. When the GAN is injured, patients may experience numbness, paraesthesia, or pain in the areas supplied by the nerve, including the lower part of the ear and adjacent skin regions. The incidence of GAN injury in such procedures has been reported to be around 6%, highlighting the necessity of precise anatomical knowledge and careful technique.

Management of GAN injuries typically involves conservative approaches, as most cases of neurapraxia (a mild form of nerve injury) tend to resolve over time without surgical intervention. However, in cases where more severe nerve damage occurs, patients may suffer from prolonged sensory deficits or discomfort. Preventive strategies include using blunt cannulas instead of sharp needles to minimize the risk of nerve damage and ensuring that threads are placed correctly within the subcutaneous layers without applying excessive tension. In more severe cases, surgical decompression of the nerve may be necessary to relieve symptoms and restore function.

In certain instances, both patients and physicians may be unexpectedly confronted with asymmetrical facial expressions following the procedure. Patients might observe difficulties with proper eyebrow elevation or noticeable asymmetry in their facial expressions when viewing themselves in a mirror with their eyes open. This asymmetry may manifest on one side of the face or affect both sides. The principal cause of this issue is frequently attributed to damage to the facial nerve during the injection and manipulation of threads, which can result in various complications. To mitigate these risks, a comprehensive understanding of the anatomical features of the lateral face is essential. The facial nerve exits below the midpoint of the parotid gland and travels beneath the SMAS layer, supplying innervation to the facial muscles. Therefore, it is crucial to ensure that threads are consistently inserted above the SMAS layer to minimize the risk of nerve damage and associated complications [17].

## 12 Parotid Gland Injury

A significant complication associated with thread lifting procedures is the inadvertent puncture of the salivary gland. This may occur if sharp needles are utilized or if the patient clenches their teeth or makes involuntary movements during the procedure, resulting in the thread perforating the gland. Symptoms generally present as unilateral swelling near the salivary gland, typically occurring between the second and seventh day following the procedure, and are often accompanied by fever and discomfort. These symptoms tend to exacerbate around mealtimes, making it essential to thoroughly evaluate them during the medical history assessment.

Research by Kim et al. [18] documented a range of symptoms in patients who sustained parotid gland or duct injuries from thread lifting, including tenderness, swelling, and pain. The severity and duration of these symptoms varied among individuals; however, all cases demonstrated improvement with conservative treatment, underscoring the effectiveness of non‐invasive management strategies. Ultrasound played a crucial role in managing these cases by enabling early detection and identification of parotid duct or gland injuries. Its use not only allowed for prompt recognition but also facilitated ongoing monitoring of the injury's progression, highlighting the importance of ultrasound in detecting and addressing delayed pain or swelling and thus reducing the risk of complications.

Treatment options include the application of pressure with a facial band, the administration of antibiotics and steroids, and the use of anti‐cholinergic medications to decrease saliva production. Additionally, the injection of botulinum toxin into the salivary gland to reduce its activity may also be beneficial [3, 18].

In rare instances, injury to the Stensen duct may occur. To prevent such complications, it is crucial to perform the procedure in a manner that avoids crossing the duct's path [19, 20].

## 13 Lateral Canthus Elevation

Elevating the lateral canthus after a procedure requires advanced techniques to achieve a natural and aesthetically pleasing result. One effective method is the temporal anchoring technique, where the tissues near the temple are secured to lift the lateral canthus. However, a common concern among patients is the risk of looking overly intense or fierce after the procedure, which can happen if the fourth thread is placed too close to the lateral canthus, specifically if the distance is less than 3–4 cm. To avoid this issue, it's crucial to maintain an appropriate distance from the lateral canthus during the procedure. Although the lateral canthus may appear slightly elevated immediately after the procedure, this effect generally subsides as the swelling diminishes within 2–3 days, returning to a more natural position. However, some patients may be particularly sensitive to even minor changes in the elevation of the lateral canthus, so careful management of patient expectations is essential to ensure satisfaction with the results [21, 22, 23].

Some younger patients may specifically seek the elevation of the lateral canthus to achieve a more defined and lifted appearance. In such cases, careful design and placement of the fourth thread can significantly increase the likelihood of a satisfactory outcome. For middle‐aged patients who are concerned about drooping of the lateral canthus or eyebrows, a slight elevation achieved through thread lifting can be an effective approach for periorbital rejuvenation. This technique can help restore a more youthful and refreshed appearance around the eyes without creating an overly intense look. In all cases, the success of the procedure depends on meticulous planning and precise execution, with special attention to thread placement to ensure a balanced and harmonious result [24].

## 14 Hematoma

Forceful or aggressive needle maneuvers can pose a risk of damaging the superficial temporal artery, potentially resulting in visible bleeding and the formation of hematomas. To mitigate this risk, it is recommended to administer an appropriate dose of epinephrine as a local anesthetic and to allow 10–15 min for its effects to take hold before beginning the procedure. By avoiding aggressive techniques during thread lifting, the likelihood and severity of hematoma formation can be significantly reduced [25].

Typically, when inserting threads, if a blood vessel is partially damaged or punctured, applying pressure to stop the bleeding is usually sufficient, and it does not pose a significant problem. However, there are cases where inserting the thread longitudinally along the direction of a blood vessel can cause a massive hematoma. This is particularly true when the thread aligns with the course of major blood vessels, such as the trunk of the superficial temporal artery or its parietal branch. Major bleeding can also occur with the facial artery and other significant facial vessels. In the forehead area, for instance, the supratrochlear and supraorbital arteries can run parallel to the thread's vector. Additionally, when inserting a volumizing thread to improve the nasolabial fold, there is a risk of longitudinally damaging the facial artery, so caution is required.

In general, the supraSMAS layer is considered a safe layer during thread lifting procedures. The blood vessels that need to be considered in this context include the superficial temporal artery and its branches, specifically the frontal and parietal branches, which are embedded in the SMAS layer. Additionally, the zygomatico‐orbital artery, which branches off from the superficial temporal artery, is another major vessel associated with the risk of hematoma. This vessel runs beneath the SMAS layer.

Moreover, when a blood vessel is longitudinally damaged, achieving hemostasis can be challenging, often leading to hematoma formation. The risk of bleeding increases when using sharp needles, whereas utilizing cannulas with a larger circumference can be beneficial in reducing this risk.

On the other side, when utilizing the deep temporal fascia for thread anchoring in lifting procedures, it is imperative to avoid the superficial temporal artery to mitigate the risk of injury. Yi and Oh [26] explored lateral facelift techniques involving thread anchoring in skin to support tissues in areas devoid of major arterial structures. Their findings indicate that longer cog threads offer superior fixation points, with optimal anchoring sites identified within the fascia of the targeted region. The study, conducted on cadaveric specimens using both micro‐computed tomography and conventional computed tomography, focused on the superficial temporal artery and its branches. The results revealed that the safest tagging area is situated within the deep temporal fascia, between the superior and inferior temporal lines. Based on their detailed anatomical analysis of the superficial temporal artery, the authors recommend this region as the most secure location for thread placement to ensure both safety and effectiveness.

## 15 Discussion

Thread lifting procedures have become increasingly popular as a minimally invasive alternative to traditional surgical methods for addressing age‐related facial changes. Despite their advantages, these procedures are not without complications, highlighting the necessity for meticulous technique and appropriate patient selection [27, 28, 29, 30, 31].

### 15.1 Immediate Complications

The most commonly encountered immediate complications of thread lifting include bruising, swelling, pain, and bleeding. Typically, these issues resolve with proper post‐procedural care. Nonetheless, more persistent complications such as dimpling, contour irregularities, and thread migration can significantly impact patient outcomes. These complications often result from improper technique, inadequate patient selection, or a lack of understanding of facial anatomy [4, 7, 32].

### 15.2 Specific Complications

Dimpling, particularly noticeable in the cheek area, is a frequent concern following thread lifting. This complication can arise from excessive tension on the threads or incorrect placement, resulting in a dimpled appearance that may necessitate corrective measures such as manual molding or additional treatments like fillers [7]. Thread migration is another challenge, where threads may shift from their intended position, potentially causing visible threads or protrusions through the skin or mucosa. This complication underscores the critical need for precise placement and stable fixation to prevent adverse outcomes and reduce the need for subsequent interventions.

More severe complications involve injury to anatomical structures, such as the parotid gland or facial nerve. Such injuries may present as swelling, pain, and asymmetrical facial expressions, necessitating careful management to minimize long‐term sequelae. Ultrasound‐guided techniques and, in severe cases, surgical interventions may be required to address these issues effectively [18].

### 15.3 Additional Risks

Hematoma formation, especially in vascularly sensitive areas such as the temporal region, is another significant risk associated with thread lifting. Employing techniques that include the use of epinephrine and precise needle manipulation is essential for mitigating these risks and ensuring patient safety during the procedure [25].

Granuloma formation and infections, influenced by thread material and insertion depth, are additional concerns. Prompt recognition and management using anti‐inflammatory medications, antibiotics, or, in some cases, thread removal, are crucial to prevent the escalation of complications and adverse impacts on patient outcomes [4, 33].

### 15.4 Future Directions

Kim et al. [18] demonstrated that ultrasound can significantly mitigate complications in thread lifting procedures, especially those involving the parotid gland. Their study, which included 703 patients undergoing facial lifting with polydioxanone threads, highlighted the effectiveness of preoperative Doppler ultrasound in accurately identifying the parotid gland and other critical structures. This early identification helps prevent injuries to the parotid duct by allowing for precise positioning before the procedure. The study advocates for routine use of ultrasound to detect and address potential injuries promptly, reducing the risk of delayed pain and other complications.

Looking ahead, advancements in imaging technologies such as high‐resolution ultrasound and real‐time 3D imaging promise to enhance thread lifting procedures further. These technologies could provide detailed visualization of facial anatomy, facilitating more accurate mapping of vital structures and minimizing inadvertent injuries. Future research should focus on refining thread placement techniques and improving thread anchoring methods to reduce common complications like dimpling and migration. Developing more precise tools for thread insertion may also contribute to greater procedural safety and effectiveness.

## 16 Conclusion

While thread lifting offers a less invasive option for facial rejuvenation, it requires careful attention to anatomical considerations, precise technique, and thorough patient management to minimize risks and achieve optimal results. Continued research and advancements in thread technology, along with ongoing education for practitioners, are vital for enhancing safety protocols and ensuring patient satisfaction. By adhering to best practices and maintaining vigilance in patient care, healthcare providers can effectively navigate the complexities of thread lifting procedures, providing patients with a safe and effective method for facial aesthetic enhancement.

## Author Contributions

All authors have reviewed and approved the article for submission. Conceptualization: Kyu‐Ho Yi, Soo Yeon Park. Writing – Original Draft Preparation: Kyu‐Ho Yi, Soo Yeon Park. Writing – Review and Editing: Soo Yeon Park. Visualization: Soo Yeon Park. Supervision: Kyu‐Ho Yi, Soo Yeon Park.

## Conflicts of Interest

I acknowledge that I have considered the conflict of interest statement included in the “Author Guidelines.” I hereby certify that, to the best of my knowledge, no aspect of my current personal or professional situation might reasonably be expected to significantly affect my views on the subject I am presenting.

## Data Availability

The data that support the findings of this study are available from the corresponding author upon reasonable request.
